# Determining the International Spread of B.1.1.523 SARS-CoV-2 Lineage with a Set of Mutations Highly Associated with Reduced Immune Neutralization

**DOI:** 10.3390/microorganisms10071356

**Published:** 2022-07-05

**Authors:** Lukas Zemaitis, Gediminas Alzbutas, Dovydas Gecys, Arnoldas Pautienius, Rasa Ugenskiene, Marius Sukys, Vaiva Lesauskaite

**Affiliations:** 1 Laboratory of Molecular Cardiology, Institute of Cardiology, Lithuanian University of Health Sciences, LT-50162 Kaunas, Lithuania; dovydas.gecys@lsmuni.lt (D.G.); vaiva.lesauskaite@lsmuni.lt (V.L.); 2 Laboratory of Translational Bioinformatics, Institute for Digestive Research, Lithuanian University of Health Sciences, LT-50162 Kaunas, Lithuania; gediminas.alzbutas@lsmuni.lt; 3 Institute of Microbiology and Virology, Lithuanian University of Health Sciences, LT-47181 Kaunas, Lithuania; arnoldas.pautienius@lsmuni.lt; 4 Department of Genetics and Molecular Medicine, Lithuanian University of Health Sciences, LT-50161 Kaunas, Lithuania; rasa.ugenskiene@lsmuni.lt (R.U.); marius.sukys@lsmuni.lt (M.S.)

**Keywords:** SARS-CoV-2, B.1.1.523, variant of concern (VOC), phylogeny, GISAID

## Abstract

Here, we report the emergence of the variant lineage B.1.1.523 that contains a set of mutations including 156_158del, E484K and S494P in the spike protein. E484K and S494P are known to significantly reduce SARS-CoV-2 neutralization by convalescent and vaccinated sera and are considered as mutations of concern. Lineage B.1.1.523 presumably originated in the Russian Federation and spread across European countries with the peak of transmission in April–May 2021. The B.1.1.523 lineage has now been reported from 31 countries. In this article, we analyze the possible origin of this mutation subset and its immune response using in silico methods.

## 1. Introduction

The emergence of severe acute respiratory syndrome coronavirus 2 (SARS-CoV-2) in late 2019 led to the ongoing Coronavirus Disease 2019 (COVID-19), now a global pandemic with more than 535 million cases of infection and more than 6.3 million deaths worldwide [1] (data from 16 June 2022). On 5 January 2020, the first whole genome sequence of 2019-nCoV was completed by the Wuhan Institute of Virology, the China Centre for Disease Control and the Shanghai Public Health Clinical Centre of Fudan University [2]. From this point on, genome sequencing has played an important role in vaccine development, and understanding its viral evolution and epidemiological characteristics. In many countries, SARS-CoV-2 sequencing has been implemented at a national level as a tool for epidemiological management [3]. 

A large dataset of SARS-CoV-2 genomes has been collected in the GISAID database, which now contains more than 3.7 million sequenced genomes from around the world [4]. As of 31 May 2021, the World Health Organization (WHO) proposed designations for global SARS-CoV-2 variants of concern (VOC) and variants of interest (VOI) to be used alongside scientific nomenclature in communications about variants to the public [5]. This list includes the variants on the global list of WHO VOC and VOI and will be updated as the WHO’s list changes. There is currently one SARS-CoV-2 VOC known as Omicron. The previous VOC’s Alpha, Beta, Gamma and Delta have been spread widely and showed significant evolutionary benefit in comparison with other lineages [6]. 

Through the routine analysis of results from the national Lithuanian sequencing efforts coordinated by the National Public Health Surveillance Laboratory, we have identified a novel SARS-CoV-2 variant classified as B.1 by PANGO but containing multiple S protein mutations associated with effects on immunity (https://github.com/cov-lineages/pango-designation/issues/69) (reported on 5 May 2021), such as E484K, 156_158del and S494P. Preliminary phylogenetic analysis indicated that this variant has a distinct viral lineage that may have originated in Russia. We reported this variant to the PANGO curators and gave it the new phylum name B.1.1.523. On 14 July 2021, the WHO added this variant to the list of variants under its monitoring section. By using bioinformatics tools, we performed a detailed analysis of this lineage and disclosed our findings about this variant and the mutational subgroups typical of this variant.

With this report, we aim to share a detailed analysis of the variant discovered, evaluate its origin as well as predict its potential epidemiological impact and risks. 

## 2. Materials and Methods

### 2.1. Collection of SARS-CoV-2 Sequences and Initial Data Processing

The sequences used for the analyses were downloaded from GISAID as of 31 August 2021 [4]. Fasta files and metadata were extracted using the nCoV-ingest tool [https://github.com/nextstrain/ncov-ingest (cloned on 19 April 2021)]. The lineages for all downloaded sequences were assigned using pangolin 3.1.11 (pangoLEARN 2021-08-24 and pango-designation v1.2.66) [7] [https://github.com/cov-lineages/pangolin (accessed on 5 May 2021)]. The general sequence quality evaluation, extraction and alignment of S protein sequences, variant calling was performed using Nextclade 1.3.020 [https://github.com/nextstrain/nextclade (accessed on 5 May 2021)] [8].

### 2.2. Transmission Cluster Analysis

In order to elucidate the potential origin of the lineage and transmission cluster, a supa phylogeny analysis of the full genomes representing a small subset of GISAID was performed. The sequences chosen for analysis were composed of the union of two sets of sequences: (i) sequences that were assigned B.1.1.523 lineage by pangolin and (ii) sequences that were at least 99.3% identical to the Latvian B.1.1.523 sequence EPI_ISL_1590462. The number of matched residues amounts to equal or more than 95% of the reference sequence. These two limits 99.3% and 95% are of a different nature. The 95% refers to the level of query coverage in the alignment despite the identity level, the aligned fraction, and the 99.3% refers to the identity level in the aligned region. The reference sequence was chosen as it was closest to the one this lineage sequenced first in Lithuania but with smaller gap regions. The alignment against the GISAID sequences was conducted using minimap2 2.20-r1061 [https://github.com/lh3/minimap2/ (accessed on 5 May 2021)] [9]. The limits were chosen arbitrarily after several attempts to look for cut-offs resulting in a set of sequences that includes most sequences from the lineage and some more diverged ones classified by pangolin as belonging to other lineages. The sequences with poor overall quality control status or with more than 1000 bps missing (as indicated by Nextclade analysis) were discarded. The maximum likelihood tree was calculated using a modified version of Nextstrain workflow [https://github.com/nextstrain/zika (cloned at 1 May 2021)]. The tree was built using IQ-TREE v2.1.2 [10]. A general time reversible model with unequal rates and unequal base frequencies was used [11] allowing for a proportion of invariable sites together with a discrete Gamma model [12]. Ultrafast bootstrap with 1000 replicates was used. The maximum likelihood emergence time and origin of country for inner nodes were calculated by treetime 0.8.1 [https://libraries.io/pypi/phylo-treetime (accessed on 5 May 2021)] as described by the aforementioned workflow. The set of sequences collected as described above was clustered into transmission clusters using Phydelity v2.0 [https://github.com/alvinxhan/Phydelity (accessed on 5 May 2021)] [13].

### 2.3. S Protein Phylogeny Analysis

The S protein-based phylogeny was based on the S protein sequences extracted from GISAID by Nextclade and aligned to the reference COVID-19 sequence. Sequences shorter than 1175 residues or having more than one stop codon or having any number of undetermined residues were discarded. Sequences were further clustered into identical sequence clusters using CD-HIT v4.8.1 [14] (command line option “-c 1.0”). The sequences representing all high-quality S protein variants were used for maximum likelihood tree calculation by VeryFastTree 3.0.1 [15] using an LG substitution model. The sequence alignment that was used to construct the tree was composed of the alignment produced by Nextclade, leaving only the representative sequences of the clusters and CAT approximation with 20 rate categories: lg -gamma. Additionally, command line flags that should increase calculation accuracy were added: “-spr 4 -mlacc 2 -slownni -double-precision”. The tree was re-rooted using sequence matching to the EPI_ISL_402124 (one of the earliest human SARS-CoV-2 sequences from Wuhan) as an out-group using GOtree 0.4.1 [https://anaconda.org/bioconda/gotree/files (accessed on 5 May 2021)] [16].

Ancestral states for inner nodes for 156, 157, 158, 484 and 494 positions of S protein were inferred using the command line version of GRASP-suite [17]. This tool for inference was chosen due to its high speed and, most importantly, ability to handle insertions and deletions. The same substitution model that was used for the phylogeny tree was also used in this case (LG). The resulting tree was analyzed and potential changes in the haplotypes were detected using a custom script written in Julia 1.6 exploiting capabilities of the NewickTree library [https://github.com/arzwa/NewickTree.jl (accessed on 5 May 2021)]. The tree was trimmed to keep only those inner nodes that lead to leaves containing the triple deletion at 156–158 positions and either E484K, S494P and visualized using ggtree 3.0.426 [18]. Additionally, a set of full genome sequences was composed matching the leaves of the aforementioned trimmed tree, and a corresponding maximum likelihood tree was calculated using the aforementioned Nextstrain workflow.

### 2.4. Analysis of Potential Recombination Events

The focused set of sequences was composed based on the sequences used for the S protein phylogeny. The set was further narrowed down to the sequences containing either the triple deletion at 156–158 positions or a combination of E484K and S494P. The detection of recombination events at the DNA level was performed using the PoSeiDon workflow [https://github.com/hoelzer/poseidon cloned at 28 September 2021] [19] that runs a GARD program to identify recombination events [20]. The genomic regions matching to the S protein were used. As the workflow is limited to 100 sequences, the initial set of 110 was clustered to 100 sequences using the cd-hit-est program from the CD-HIT package 4.8.1 using identity cut-off equal to 0.9998658. The detection of recombination effects at the protein level was performed using the detREC tool [https://github.com/qianfeng2/detREC_program (cloned on 1 October 2021)] [21]. The recombination detection was conducted using either a genomic or protein sequence corresponding to the S protein. The full genome scale recombination analysis was performed for the same set of 110 sequences using the 3SEQ program [22].

### 2.5. Antibody Escape Effect Estimation

An escape effect against an antibody targeting the NTD domain was evaluated using structural analysis [23]. In order to evaluate the escape effect for S protein, four sequence variants were tested: (i) the wild type, (ii) 156_158del (B.1.1.523 lineage), (iii) 156_157del & R158G (Delta variant), and (iv) S protein sequence with multiple mutations matching NCBI Genbank entry UFO69279.1 (Omicron). These sequence variants were modelled using part of the 7LQV structure as a template: 14-283 residues from a chain A (spike protein), 1-113 residues from a chain H (antibody) and 1-106A residues from a chain L (antibody) employing Rosetta package (2021.16.61629_bundle) [24]. The S protein residues from 284 position to the “N” terminus were discarded. The PDB structures for fragment library generation and other required databases for Rosetta were downloaded at 2 June 2021. The S protein structure models were created using a comparative modelling approach following guidelines in the documentation. During the homology modelling Rosetta runs, the antibody structure including side chains was kept constrained using a Coordinate Constraint Generator. For the relaxation part, an “InterfaceRelax2019” script was used. The lowest energy model was chosen from 200 models using two applications from the Rosetta package: (i) clustering with an energy-based_clustering application, and (ii) choosing the best scoring cluster centroid using the score_jd2 application.

The antibody binding for the chosen model was further evaluated performing local docking in two ways (i) using HADDOCK 2.2 [25], and (ii) using the docking protocol application from the Rosetta package [24] in combination with scoring by PRODIGY [26,27].

HADDOCK 2.2 was run via the Refinement interface on the webpage (https://milou.science.uu.nl/services/HADDOCK2.2/haddockserver-refinement.html, (accessed on 12 June 2022) using the default parameters. 

The antibody docking was also carried out using the docking-protocol application from the Rosetta package. At first, local docking was run using “-partners LH_A -ex1 -ex2aro -dock_pert 3 8” non-default flags, then the refinement step was conducted using “-partners LH_A -use_input_sc -docking_local_refine -ex1 -ex2ar” non-default flags. The resulting complex structure was scored with the InterfaceAnalyzer application using “-interface LH_A -pack_separated” non-default flags [24]. For each model, docking was run 5000 times and the top 500 structures were chosen for analysis based on the interface score (I_sc). The complex formation energy of the 500 structures for each sequence variant was predicted using PRODIGY [26,27] command line program (https://github.com/haddocking/prodigy, master branch cloned on 20 June 2022). The binding was evaluated using the non-default flag “--selection H, L, A” (this requests to evaluate interaction between the antibody (H,L chains) and the S protein).

Additionally, the four homology models (starting structures for docking rins) were evaluated using the PRODIGY program via the web interface (https://wenmr.science.uu.nl/prodigy/ (accessed 12 June 2022)) [27]. The RMSD between the docked structures resulting from the calculations by the docking protocol application from the Rosetta package and the homology models (starting structures) were calculated using the PyMOL 2.5 [28] command line interface via the “align” command.

The antibody escape effect of E484K and S494P mutations was analyzed using the FoldX 4 program [26]. The antibody structures targeting the receptor binding domain of the S protein were downloaded from CoV3D as available on 1 May 2021. The structures were initially processed by the pdb2pqr v3.1.0 [https://doi.org/10.1002/pro.3280] program using these non-default command line flags: “pdb2pqr30 --drop-water --ff AMBER --keep-chain --pdb-output”. The mutations into the complexes were introduced and binding energy was calculated by consecutively applying the FoldX commands: RepairPDB, BuildModel, and AnalyseComplex. The residues at 484 and 494 positions were modelled either to the residue matching the mutation or to the wild type residue, and changes in free energy upon binding were evaluated. The AnalyseComplex command was run using a non-default flag “—analyseComplexChains = A”.

The synergy of the escape effect was evaluated as follows. At first, for a given complex structure, relative ∆∆G was calculated using the FoldX output using this formula:

$$∆∆G_{\mathrm{mutant}\;\mathrm{relative}}=\frac{∆G_{\mathrm{mutant}}-∆G_{\mathrm{wildtype}}}{\left|∆G_{\mathrm{wildtype}}\right|}$$

where ∆G is the free energy of the complex. The higher the ∆∆G, the greater extent to which the binding to an antibody was disturbed due to a mutation. For example, if ∆G_wildtype_ is −10 kcal/mol and ∆G_E484_ is −5 kcal/mol, then the ∆∆G_E484_ would be (−5–−10)/10 kcal/mol, which is 0.5 or 50%. The relative, and not the absolute value, was used to make the evaluation robust to variations in absolute ∆G values and make results comparable across different complexes. 

The synergy of the double E484K & S494P mutant was evaluated by introducing a relative ∆∆∆G that was calculated using this formula:

$$∆∆∆G_{\mathrm{relative}}=\min\left(∆∆G_{E484\&S494P\;\mathrm{relative}}-∆∆G_{\mathrm{mutant}\;\mathrm{relative}}\right),\;\mathrm{mutant}\in \left\{E484K,S494P\right\}$$

For example, if ∆∆G_E484_ is 0.2, ∆∆G_S494P_ is 0.4 and ∆∆G_E484&S494P_ is 0.6, then the corresponding ∆∆∆G_relative_ would be min((0.6–0.2), (0.6–0.4)), which is 0.2 or 20%. The greater the relative ∆∆∆G, the greater is the escape synergy, or in other words, the greater is the disruption of a complex by the double mutant compared with any of the single point counterparts.

## 3. Results

### 3.1. Mutation Review of B.1.1.523

**Figure 1:**
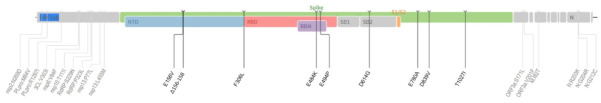
Mutation overview in the B.1.1.523 lineage. Several other mutations have been observed in the spike-protein sequence of the B.1.1.523 variant, including E156V, F306L, D614G, E780A, D839V and T1027I.

Several mutations in the S region have been observed in the B.1.1.523 variant, from which 156_158del, E484K and S494P are considered as an attribute of VOM (Figure 1). According to previously reported data, the 156_157del and G158R mutations in the Delta variant match the same protein surface area as the 144 and 241–243 deletions in the Alpha and Beta (B.1.351) variants, respectively. These altered residues are found in the NTD ‘supersite’ targeted by most anti-NTD neutralizing antibodies, thus providing a mode to evade the immune system [29]. Moreover, the E484K mutation also contributes to SARS-CoV-2 immune system evasion. Several recent studies have observed that E484K may significantly reduce convalescent serum neutralization [30,31]. Additionally, it was observed that the S494P mutation is related to a 3–5-fold reduced SARS-CoV-2 neutralization in sera. With the combination of the 156_158del, E484K and S494P mutations, as shown in Figure 1, the B.1.1.523 lineage should remain on epidemiologists’ watchlists as one of the concerning SARS-CoV-2 lineages. Moreover, the T1027I substitution in the spike is in a perfectly conserved amino acid strand [32] typical of the former VOC gamma variant (P.1). We cannot find any studies focused on investigating the effect of this mutation. E156V, F306L, E780A and D839V are not typical in other lineages, but are observed in Delta, Omicron, Gamma and other lineages. The total number of sequences published in GISAID with these mutations (13 June 2022) is 615, 2540, 962, and 832, respectively, out of a total of 11.4 million published genomes.

### 3.2. The Origin and Formation of Key S Protein Formation of the Lineage

**Figure 2:**
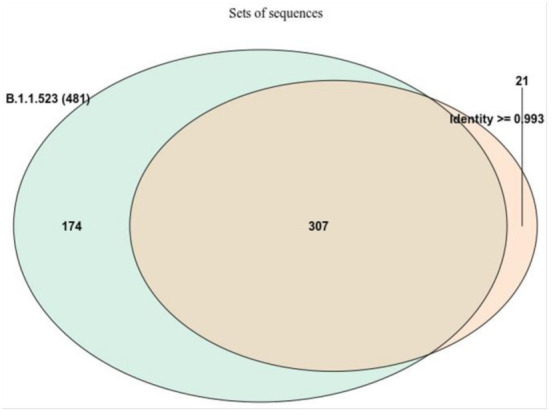
The overlap between the two data sets used for the focused ML tree. The sequences were chosen either based on the Pango assignment or by identity with a B.1.1.523 lineage sequence (EPI_ISL_1590462). Most of the sequences with high identity (>0.993) to the Latvian B.1.1.523 lineage were classified as belonging to B.1.1.523. However, 21 sequences (6%) were not assigned to B.1.1.523.

One of the objectives of the analysis was to determine if the two clusters of mutations, which are responsible for immunity resistance, were acquired by sequential mutations or if this is a result of recombination events. Initially, a list of data entries comprised of the sequences classifiable as B.1.1.523 lineage together with the closest sequences based on identity was used to construct a maximum likelihood (ML) tree. The size and overlap between the two data sets is depicted in Figure 2.

The 21 sequences matched only by identity were mostly assigned by pangolin to the B.1/B.1.1 lineages. The generation of ML revealed several interesting properties of B.1.1.523 (Figure S1). At the base of the lineages leading to B.1.1.523, sequences with a full set of expected S protein mutations branch away in clusters of sequences which have the triple S:156–158 deletion. The sequences which have the additional substitutions at S:484 and S:494 positions emerge further in the evolution. However, here, we have no clear indication about whether the mutations S:E484K and S:S494P are introduced sequentially from the B.1.1.523 lineage.

We have observed that some sequences which originated from progenitors with a full set of expected substitutions at 484, 494, 156, 157 and 158 S region positions underwent a reverse-type mutation to wild type variants. Such events are highly unlikely and are usually caused by erroneous sequence assembly or could be an indication of low-quality data. 

**Figure 3:**
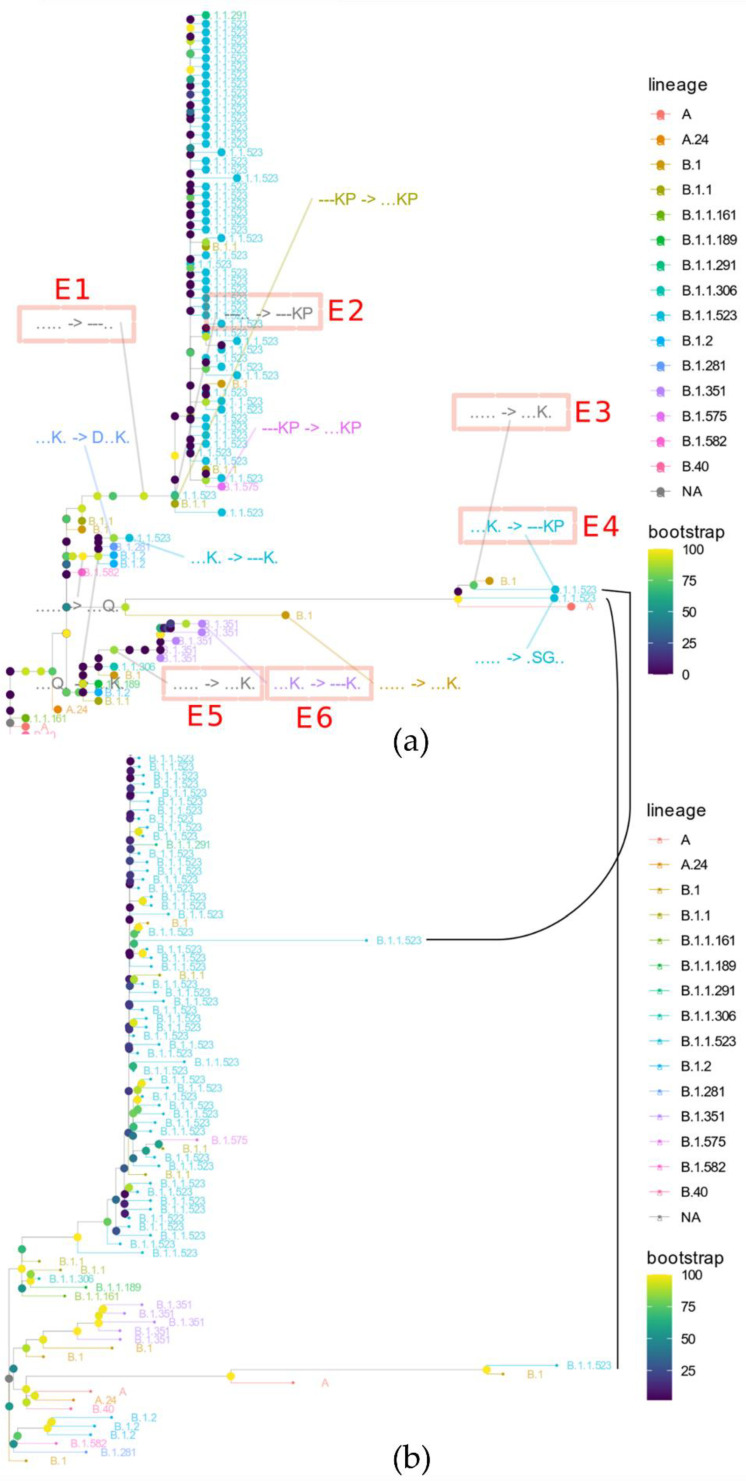
Phylogeny based on S protein sequence. The color of the dots denoting inner nodes depicts the bootstrap values. (**a**) The tree represents a maximum likelihood tree based on all unique S protein sequences of the genomes deposited into GISAID. The visible subset of the tree matches lineages that lead to branches which have 156_158del and E484K or S494P mutations. The arrow “->” indicates haplotype transitions detected by comparing parental sequences with their offspring variants. The five-letter haplotype strings match 156, 157, 158, 484 and 494 positions of the S protein with “.”, meaning the wild type. The pink squares and the labels in red indicate prominent transitions discussed in the main text. (**b**) The lower tree matches the maximum likelihood tree based on whole genomes of the cases visualized in the upper tree. The black solid lines indicate a match of nodes for two B.1.1.523 sequences from Turkey in the phylogenies based on the S protein and whole genome.

Additionally, to discern the cases in which the mutations comprised of the two regions of potential enhanced resistance to immune response have been combined, phylogeny analysis of all the unique S protein variants was performed. As depicted in Figure 3, there are at least three distinct cases in which E484K and/or S494P have been combined with 156_158del. The visualized bootstrap values were calculated either by the default method used by VeryFastTree (Figure 3a) or IQ-TREE (Figure 3b), as indicated in Section 2. The phylogeny analysis of the S protein indicates that lineage assignments by Pango can change and sometimes are not stable across different Pangolin versions when pangoLEARN is used [33]. Therefore the assignments can be misleading and relying on the plain assignments could hide novel developments of SARS-CoV-2. For example, some sequences from Turkey that were assigned as lineage B.1.1.523 originated from distinct clusters based on S sequence (Figure 3a) and were evidently evolutionarily distant from other B.1.1.523 lineage genomes.

Transitions E1 (introduction of the triple deletion) and E2 (introduction of E484, S494P) (Figure 3a) indicate a pathway which the majority of B.1.1.523 lineage sequences took. As in the case of data submitted, we do not detect sequential acquisition of E484K or S494P, and immediately next to the triple deletion, we see the two additional mutations. This could indicate a potential recombination event but it is most probably due to lack of sufficient data. 

**Figure 4:**
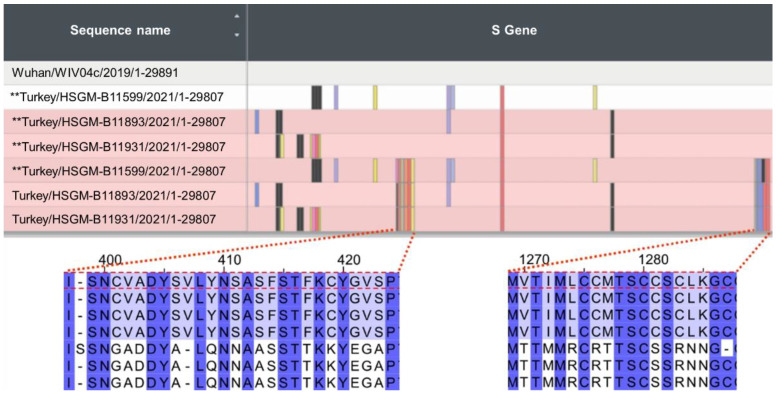
S protein region of B.1.1.523 sequences from Turkey. Turkey/HSGM-B11599/2021 and Turkey/HSGM-B11931/2021 were classified by Pango as belonging to the B.1.1.523 lineage. The top figure is a snapshot from the Nextclade analysis. The top sequence is the reference sequence hCoV-19 used by GISAID. The asterisks ** indicate a sequence from Turkish variants that have their two most variable sequences manually swapped with corresponding regions from the reference sequence. The two lower graphs indicate the sequence alignments from the two extremely variable fragments. The order of sequences is the same as in the upper graph: reference sequence, three Turkish sequences with swapped fragments and three original Turkish sequences.

An interesting case is observed with two highly diverged Turkish sequences classified by Pango as B.1.1.523 lineage. In this case, following the most likely scenario, the E484K was acquired first and only then were the S494P and E156_R158del acquired (Figure 4). As in the case discussed above, the sequential steps acquired are missing from the data. Most probably, the sequencing data was not comprehensive enough to reveal the full picture of how the combination was formed. 

**Figure 5:**
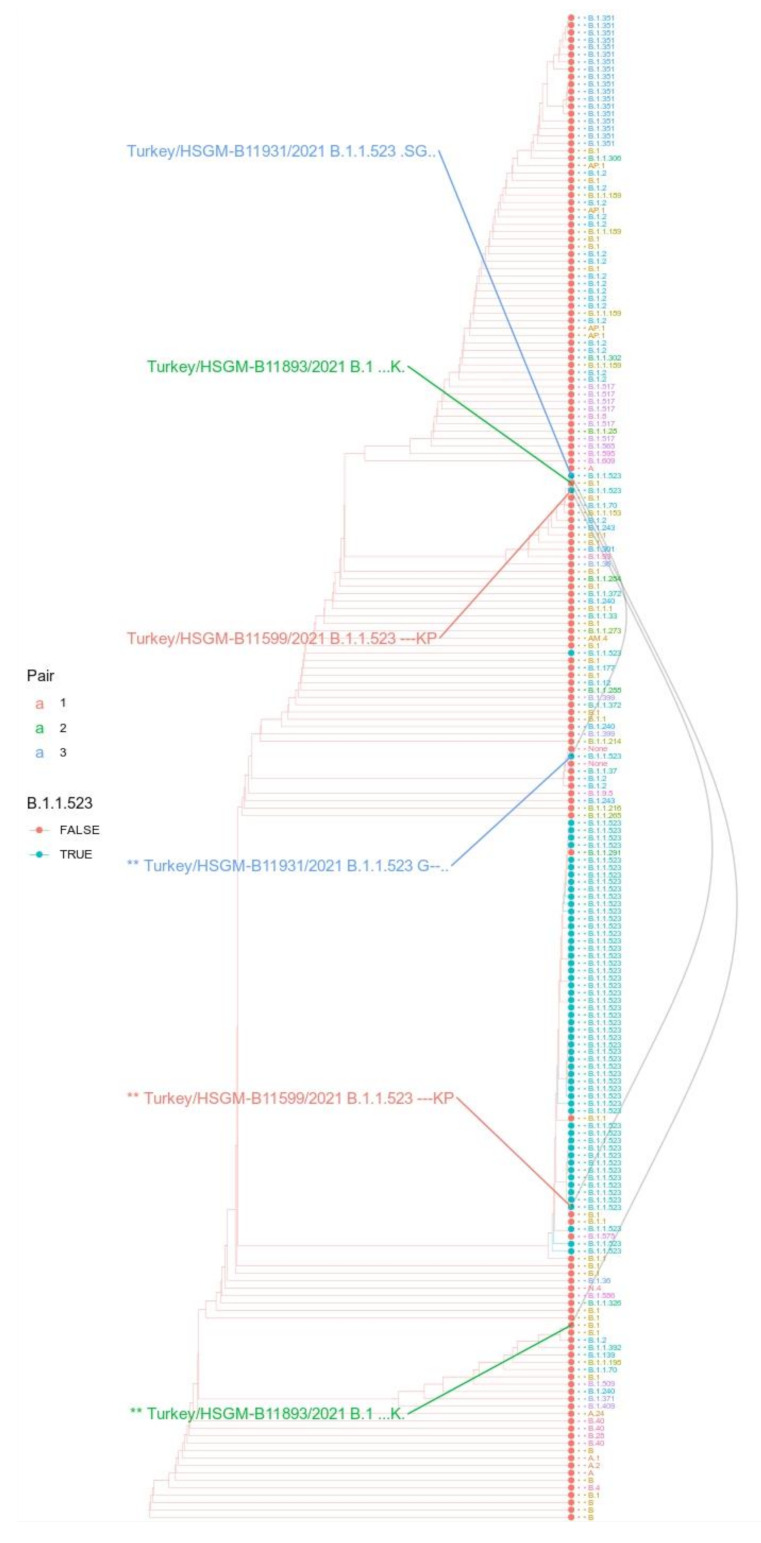
Phylogeny based on S protein sequence with modified sequences from Turkey. The cladogram of the maximum likelihood tree includes the three pairs of Turkish sequences with their variable regions either swapped with counterparts of reference sequences or left as original. The color of the tips of leaves indicates if they are classified as B.1.1.523. Tip labels indicate their Pango assignment with colors indicating different lineages. The different tips connecting grey lines indicate a Turkish sequence. The asterisks (“**”) in front of the sequence name labels indicate the sequence variant where the variable regions were swapped with corresponding regions from the reference sequences. Next to the sequence labels, the haplotype at positions 156–158,484,494 with “.” indicates the wild type and “-” a gap.

In addition to these two sequences assigned to the B.1.1.523 lineage, one Turkish sequence was placed in the ML tree immediately next to them (Figure 5) but was assigned to the B.1 lineage. For each of the three sequences, we created a synthetic sequence with the variable fragments switched to the wildtype match (Figure 4, asterisks denote the synthetic variants). These two highly diverged Turkish B.1.1.523 sequences indicate that there are vast uncharted territories in COVID-19 evolution, as we have only sparse sequencing data obtained from central Asian, African, southeast Asian, and east Mediterranean regions where no more than 1.6% of confirmed cases are sequenced [34]. An evident third case emergent combination of immune response hindering mutations from distinct S protein regions is highlighted by E5 and E6 in Figure 3a. In this case, within the B.1.351 lineage, the E484K mutation was introduced first and then E156_R158del followed. The data presented shows that, evidently, the immune response-hindering mutations from the RBD and NTD domains have been combined in one protein at least three times. In two of them, E484K occurred first; in one case, the first one was the triple deletion. Most probably, these are the results of independent mutational events.

The Turkish sequences assigned to the B.1.1.523 lineage depicted in Figure 5 are highly diverged from the wild type based on the regional variation in the S protein. According to the GISAID Audacity Instant search (performed on 7 October 2021) [4], the first variable region (bottom left, Figure 5) from Turkey/HSGM-B11599/2021 was found in seven sequences, and the second variable region (bottom right, Figure 5) was found in four sequences. The sequences have been deposited into GISAID during several submissions. Other genomic regions of the Turkish sequences shown in Figure 4 do not contain such large SNP clusters as the ones shown in the S protein. This is indicated by the changes in the placement of the three Turkish sequences upon switching the two extremely deviated fragments with corresponding fragments with the reference sequence (the black lines in Figure 5 connect the original sequence variant with the manually changed sequence). The switch to the wildtype fragment for the three sequences changes the placement and they are no longer clustered close to each other. Only one of them after the switch is clearly placed together with other B.1.1.523 sequences. 

### 3.3. B.1.1.523 Spread Worldwide

**Figure 6:**
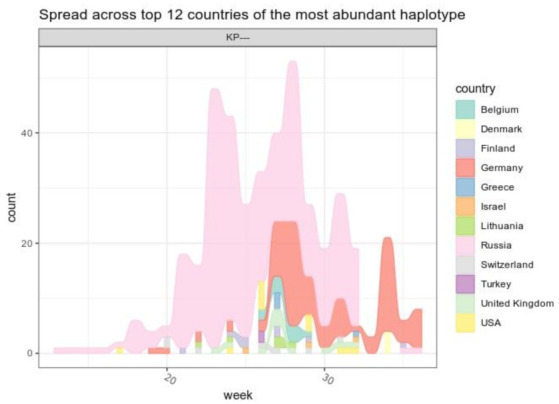
The distribution of cases of the lineage B.1.1.523 across countries at different time points. The “0” time point indicates the date of the earliest lineage sequence uploaded onto the GISAID database. Only sequences that have the typical set of S mutations were considered (E484K, S494P, 156_158del). Only the cases which correspond to the top 12 countries with the most abundant detection rate are included in the underlying data. The top 12 countries correspond to 93% of all cases.

**Figure A1:**
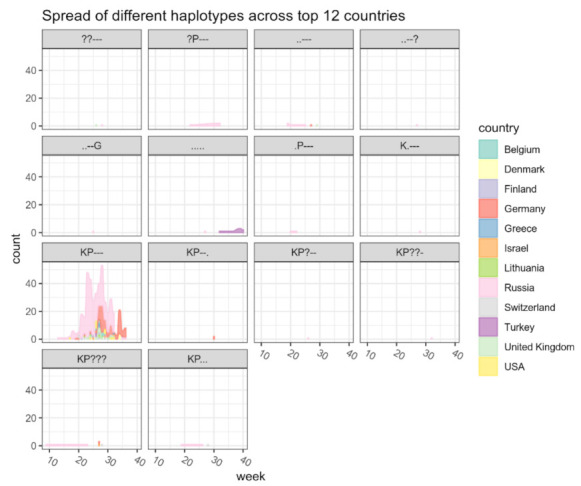
The distribution of cases of the lineage B.1.1.523 and different haplotypes based on five positions across countries at different time points. The “0” time point indicates the date of the earliest lineage sequence uploaded onto the GISAID database. The haplotypes for 484,494,256,257,258 S protein positions are indicated in the gray background (“.”—wild type residue, “?”—undetermined haplotype). Only the cases of B.1.1.523 which correspond to the top 12 countries with the most abundant detection rates are included in the underlying data. The top 12 countries correspond to 93% of all cases.

Until 31 August 2021, 459 B.1.1.523 sequences spread across 31 countries have been published in the GISAID. According to the analysis results, B.1.1.523 originated in the Russian Federation and spread across European countries (Figure 6). The sequenced clades peaked at week 25 and then subsided. The B.1.1.523 variant with the set of all five typical mutations at the five positions was the dominant one (Figure A1). In total, 95 transmission clusters have been identified. The peak of B.1.1.523 transmission intensity was around April–May 2021. The most numerous transmission clusters were detected with MRCAs originating from Germany and Russia.

**Figure 7:**
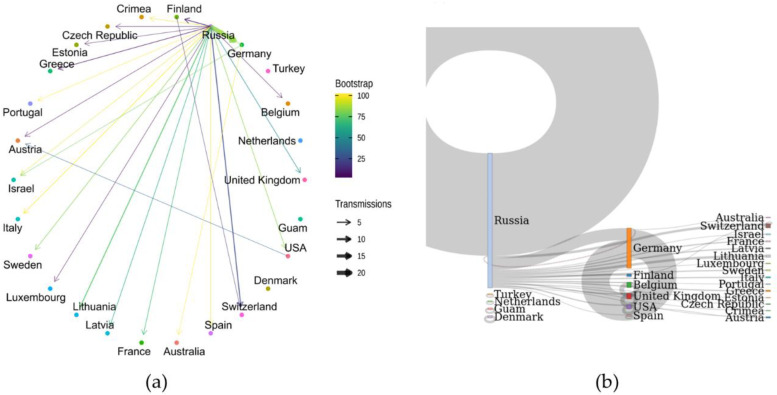
B.1.1.523 Transmission clusters. Sequences with identity larger or equal to 99.3% of the Latvian B.1.1.523 sequence (EPI_ISL_1590462) were used for the analysis. (**a**) The plot indicates the number of cases where the assigned country to a node of the most recent common ancestor (MRCA) of a cluster is different from a sequence that belongs to the cluster. The arrow starts at a country name that matches a MRCA node of clusters (origin) and it points to a country matching a country of a leaf (destination). The color of the edges denote median values of the ultrafast boot strap approximation values for the corresponding MRCA node. (**b**) The corresponding Stankey diagram of visualizes cases with the same origin and destination.

**Figure 8:**
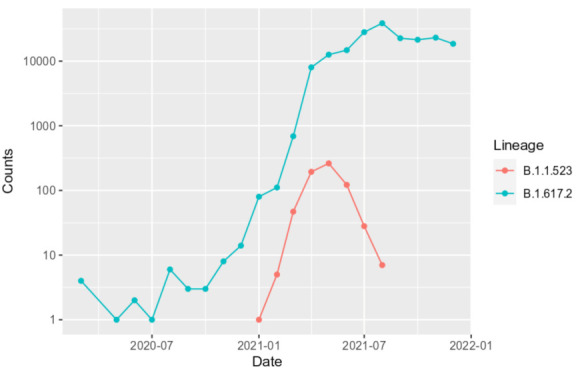
Newly sequenced cases of B.1.1.523 and Delta lineages (B.1.617.2) in Germany. The data number of new cases per month and lineage assignments are based on GISAID metadata (parsed on 20 May 2022). Counts represent number counts per month based on the date attribute of the GISAID data. Only sequences defined to a day value in the date were considered.

In most of the cases, the transmission origin country (country inferred for a cluster MRCA sequence) and the target country (country of a sequence indicated in the GISAID metadata) was Russia (Figure 7). As we see in Figure 7b, most of the transmission events occurred within Russia. Evidently, the largest number of cases where transmission origin and target countries were different resembles transmission from Russia to Germany. The data indicates one reverse type (Germany to Russia) transmission. As of the date this article was written, Germany can be considered as a reservoir of the B.1.1.523 lineage. In May 2021, the B.1.1.523 and Delta variants represented 0.002% and 0.054%, respectively, whereas the data for August 2021 shows a steady increase in detected cases to 0.327% and 32.358%, respectively. The overall number of sequenced cases in Germany is overwhelmed by more contagious the Delta (Figure 8) and Omicron subtypes. However, the B.1.1.523 lineage does not seem to be fade away until August 2021. According to GISAID (13 June 2022) data, the current predominant virus variants in Germany and most of the other B.1.1.523 affected countries are the Omicron subtypes, which have prevailed over B.1.1.523 and the Delta variants. As indicated in Figure 6, the spread of the lineage (based on GISAID submissions) diminished in Russia while it circulated widely in Germany until August 2021. It is unclear whether B.1.1.523 is completely out of circulation in Russia or its frequency dropped below the sensitivity threshold of SARS-CoV-2 genomic surveillance in this country.

### 3.4. B.1.1.523 Antibody Escape

**Figure 9:**
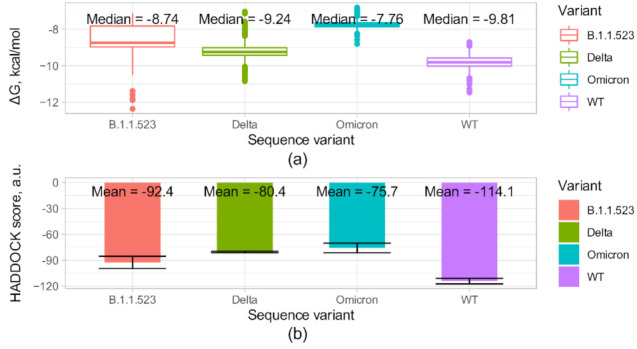
Escape effects of the d156_158 mutation (B.1.1.523), del156_157&R158G mutations (Delta variant), and d130_d132&d56_57 mutations (Omicron) on NTD-directed neutralizing antibody 4–8 Fab [23,36]. (**a**) Complex formation energy predicted by PRODIGY [27] based on top scoring structures resulting from local docking. For all possible pairs of sequence variants, *p* values for pairwise comparisons using Wilcoxon rank sum test with continuity correction were <2 × 10^−16^. The *p* values were corrected for multiple comparisons using the Benjamini–Hochberg procedure. (**b**) HADDOCK docking scores in arbitrary units (a.u) for the mutants and the wild type complexes. The error bars represent the standard deviation.

**Figure A2:**
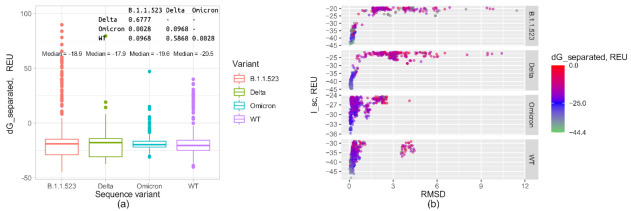
WT Omicron Rosetta local docking results modelling escape effects of the d156_158 mutation (B.1.1.523), del156_157&R158G mutations (Delta variant) and d130_d132&d56_57 mutations (Omicron) on NTD-directed neutralizing antibody 4–8 Fab [23,36]. (**a**) Complex formation energy represented by Rosetta dG_separation score of top scoring structures resulting from local docking. The *p* values for pairwise comparisons using Wilcoxon rank sum test with continuity correction are indicated in the top right corner. The *p* values were corrected for multiple comparisons using the Benjamini–Hochberg procedure. (**b**) RMSD values (comparing to the initial structure) and dG_separation scores for the 500 top scoring structures from the docking calculations (chosen out of 5000 based on the I_sc score).

In Figure 9a, the predicted free energy of complex formation for the three sequence variants are presented considering the NTD-antibody complex. The ∆G of the complex formation was predicted using the PRODIGY [27] program based on the 500 top scoring structures resulting from the Rosetta docking runs for each sequence variant. The PRODIGY scores correlate well with experimentally determined binding ∆G [27]. The data could allow ranking of the sequence variants in terms of decreasing affinity as follows: WT < Delta < B.1.1.523 < Omicron. The matching data on Rosetta dG_separated scores and RMSD values for the 500 structures for each sequence variant are presented in the Figure A2. The analogous docking using HADDOCK [25] is presented in Figure 9b. The HADDOCK scores positively correlate with experimental binding free energies [35], and in terms of decreasing mean score of the best cluster, the sequence variants could be arranged in the following order: WT < B.1.1.523 < Delta < Omicron. Both methods indicate that the Omicron variant disrupts the antibody binding to the largest extent. In addition, both methods predict lower affinity to the antibody for B.1.1.523 and Omicron sequence variants compared with the wild type. These differences were shown to be statistically significant by pairwise comparisons using the Wilcoxon rank sum test analyzing the PRODIGY predictions (Figure 9a). 

**Figure 10:**
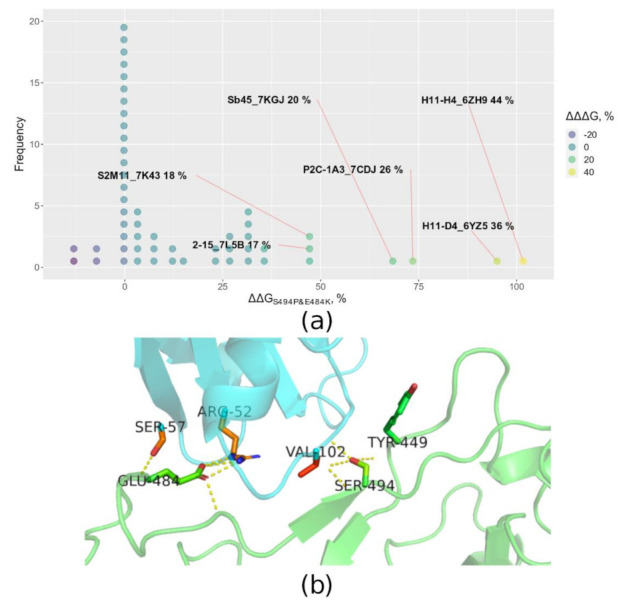
Escape effects of the E484 and S494P mutations and their combination. (**a**) The distribution of relative ∆∆∆G values based on FoldX calculations using available S protein—antibodies crystal structures. The ∆∆G values indicate a relative increase in binding energy compared with the wild type structure for a particular mutation or set of mutations. The ∆∆∆G indicates the minimum difference between the ∆∆G of the double mutation and any of the two single point mutations. The larger the value, the larger the synergy in evading interaction with the antibody. If a complex’s ∆∆∆G was greater than 15%, its label is given containing the name followed by the corresponding value of ∆∆∆G. (**b**) The structure of an antibody and receptor binding domain of the S protein complex (PDB ID: 6YZ5 that was highly impacted by the double mutation [38]. The green color depicts the S protein, cyan color—antibody chain. Sticks depict the E484 and S494 residues together with residues that form polar contacts with E484 or S494. The polar contacts are depicted by yellow dashes.

All calculated FoldX binding energies are given in the Supplementary File FoldX_data.zip. The csv file provided contains data on binding energies of the three mutants (two single point and one double) and the corresponding wild type structures. As described in Section 2, the synergy of the two mutations was evaluated using a relative measure ∆∆∆G. The relative values were used in order to make the evaluation robust to variations in absolute ∆G values and make the results comparable across different complexes. The relative ∆∆G values for single point mutants and the corresponding ∆∆∆G values for the cases with the highest values of the latter are shown in Figure 10a [37].

As we see in Figure 10a the majority of analyzed complexes are not influenced by the combination of the two mutations. As we see from the ∆∆G_S494P&E484K_ values, apart from the complexes that should be disrupted by the combination of the two mutations, there are complexes that should be stabilized, as indicated by negative ∆∆G values. The complexes that have the highest ∆∆G_S494P&E484K_ (the highest complex disruption by the double mutation) are also prominent for the highest ∆∆∆G values that indicates synergy. 

It is interesting to notice that the two most disrupted complexes are single chain synthetic antibodies from structures 6YZ5 and 6ZH9 [38]. However the isolated monoclonal antibodies from structures 7KGJ [39] and 7CDJ [40], along with an antibody from structure 7K43 [41], are also highly impacted.

## 4. Discussion

We have identified a new SARS-CoV-2 virus lineage with multiple mutations associated with immune escape and reported this to Pango on 5 May 2021 [https://github.com/cov-lineages/pango-designation/issues/69], which mandates the new lineage designation B.1.1.523. This lineage was first determined in March 2021, and at the time of writing this article, the total number of cases had reached 623 across 31 countries [42]. It is likely that the rapid increase in the circulation of the Delta variant could have diminished the rise of the B.1.1.523 lineage. The spread of the B.1.1.523 SARS-CoV-2 lineage was pushed out by other lineages, and the last case was observed in a sample from France, collected in 31 August 2021 (EPI_ISL_4412356). Interestingly, the transmission of this lineage has diminished in Russia, where it was most expected to rise. This can be explained by different diagnostic strategy approaches in the Russian Federation, where the testing is performed on non-randomly selected sources in the country. Alternatively, this could be explained by the steep rise in the Delta variant in Russia, which started a month earlier than in Europe. For example, in mid-June, more than 80% of cases in Russia were Delta, whereas in Germany the same frequency of Delta was observed only in mid-July.

The B.1.1.523 lineage possesses three or more mutations that characterize SARS-CoV-2 VOCs, including the S:D156-158 deletion, S:E484K and S:S494P. The D156-158 deletion is at the β-hairpin antigenic supersite, located in the same region typical for the Delta variant (E156G and 157-158del) [43]. The E484K mutation has been detected in the previous VOC Beta variant (B.1.351) and VUM Zeta (B.1.1.28). The mutation is in the genomic region coding the SARS-CoV-2 spike protein and it appears to have a significant impact on the body’s immune response and, possibly, vaccine efficacy. On 1 February, Public Health England (PHE) announced that the COVID-19 Genomics (COG-UK) consortium had identified this same E484K mutation in 11 samples carrying the Alpha variant B.1.1.7, after analyzing 214,159 sequences [44]. An in vitro study for the SARS-CoV-2 spike protein mutations responsible for antibody evasiveness has identified that the S494P mutation reduces SARS-CoV-2 neutralization by 3–5-fold in some convalescent sera [45]. Additionally, Lassaunière et al. found that lineages with E484K showed a significant reduction in virus neutralization titers relative to D614G (4.0-fold) and other Delta strains tested, including B.1.617.2 (2.3-fold), AY.4 (2.3-fold), and AY.4.2 (1.7-fold) [46]. Li, et.al observed that additional S494P showed increased Alpha and Beta variants infectivity [47]. Our analysis indicates that there is a possibility of antibody escape synergy for the S:E484K and S:S494P combination. The potential synergetic effect was most evident for the single chain antibodies; however, our modeling showed that it can also be substantial and also in the cases of monoclonal human origin antibodies. Sequences currently deposited into GISAID do not support the hypothesis that S:156_158del was combined with S:E484K and S:S494P during a recombination event. Instead, it appears that the triple deletion was introduced initially, and then the addition of S:E484K and S:S494P followed. 

We have shown by molecular modelling that in at least one antibody case the triple deletion del156-158 could decrease interaction. The combination with other immune escape enhancing mutations at RBD could result in a variant highly resistant to immunity formed from initial virus variants. The Delta variant also posseses sequence changes at the S protein residues 156-158 that can induce immune escape and recombination with the B.1.1.523 variant or de novo introduction of the E484K and S494P mutations could make the variants more contagious. It is worth noting that a Delta variant containing the E484K mutation has already emerged [48].

Our modeling showed that, at least in the case of one NTD targeting antibody, an Omicron sequence variant is notable as having the lowest potential affinity, followed by those for Delta and B.1.1.523. Two docking approaches were consistent in indicating that the Omicron sequence variant disrupts the interaction with the antibody to the largest extent compared with the other three sequence variants. Additionally, both methods showed that the B.1.1.523 and Delta variants should also bind the antibody with lower affinity compared with the wild type. This is clearly consistent with experimental data showing that the Omicron variant is capable of immune escape [49] and could partially explain why B.1.1.523 did not spread: Omicron could have an advantage in escaping antibodies targeting the NTD domain of the S protein. 

The case with the Turkish sequences is controversial. The sequences in the S region significantly deviated fragments with many “private” mutations. These sequences have been seen across several submissions. Hence, these might not be artificial. If these sequences are not artefacts, then the Turkish sequences classified by Pango as belonging to the B.1.1.523 lineage resulted after a recombination event between a highly diverged variant from Turkey and a typical B.1.1.523 lineage sequence. In Turkey, the virus has spread widely with limited control and reporting [50]. Therefore, widely divergent variants may have evolved. The results indicate that the Pango classification should not be taken for granted. Of the two Turkish sequences assigned to the B.1.1.523 lineage, only one was characteristic of the lineage SNPs at the S protein; the other one (EPI_ISL_2403346) has mutations characteristic of the Delta variant (a double deletion and G residue in the 156–158 region). This issue was resolved in Pango v.4.0.6 PLEARN-v1.8 version.

## 5. Conclusions

Although this variant no longer poses a risk and has been displaced by other VOCs, the study of a combination of mutations in the B.1.1.523 lineage has shown a synergistic effect in antibody escape, which may be useful for understanding new variants of SARS-CoV-2 and the evolution of mutations.

## Data Availability

The datasets analyzed during the current study are available in the GISAID and COV3D repositories, the corresponding persistent links: https://www.gisaid.org/ (accessed on 5 May 2021) [4] and https://cov3d.ibbr.umd.edu/ (accessed on 5 May 2021) [51]. All data generated during this study are included in this published article and its Supplementary Information Files.

## Supplementary Materials

The following supporting information can be downloaded at: https://www.mdpi.com/article/10.3390/microorganisms10071356/s1, Figure S1: Maximum likelihood tree based on the genomic sequences that are classifiable as B.1.1.523 lineage together with the closest sequences based on identity search; File S1: FoldX binding.

## Appendix A

**Table A1:** The Prodigy analysis results of homology models of complexes comprising the S protein N-terminal domain targeting antibody and S protein variants. The 7LQV (PDB ID) structure was used as a template.

		Sequence Variant			
		WT	B.1.1.523	Delta	Omicron
	Predicted binding ∆G, kcal/mol	−11.2	−9	−9.6	−8.8
Number of Interfacial Contacts (ICs)	ICs charged-charged	8	3	5	3
	ICs charged-polar	9	7	9	8
	ICs charged-apolar	14	6	7	17
	ICs polar-polar	2	3	4	4
	ICs polar-apolar	14	12	14	7
	ICs apolar-apolar	14	14	17	9
Non-Interacting Surface (NIS)	NIS charged,	17.95	17.81	17.86	18.73
	NIS apolar,	38.72	39.95	39.8	39.49

## References

[1] World Health Organisation WHO Coronavirus (COVID-19) Dashboard. https://covid19.who.int/.

[2] Wu F., Zhao S., Yu B., Chen Y.M., Wang W., Song Z.G., Hu Y., Tao Z.W., Tian J.H., Pei Y.Y. (2020). A new coronavirus associated with human respiratory disease in China. Nature.

[3] European Centre for Disease Prevention and Control How ECDC Collects and Processes COVID-19 Data. https://www.ecdc.europa.eu/en/covid-19/data-collection.

[4] Yuelong S., John M. (2017). GISAID: Global initiative on sharing all influenza data—From vision to reality. Eurosurveillance.

[5] World Health Organisation Tracking SARS-CoV-2 Variants. https://www.who.int/en/activities/tracking-SARS-CoV-2-variants/.

[6] European Centre for Disease Prevention and Control SARS-CoV-2 Variants of Concern as of 30 September 2021. https://www.ecdc.europa.eu/en/covid-19/variants-concern.

[7] O’Toole Á., Hill V., Pybus O.G., Watts A., Bogoch I.I., Khan K., Messina J.P., Tegally H., Lessells R.R., Giandhari J. (2021). Tracking the international spread of SARS-CoV-2 lineages B.1.1.7 and B.1.351/501Y-V2. Wellcome Open Res..

[8] Aksamentov I., Roemer C., Hodcroft E.B., Neher R.A. (2021). Nextclade: Clade assignment, mutation calling and quality control for viral genomes. J. Open Source Softw..

[9] Li H. (2021). New strategies to improve minimap2 alignment accuracy. Bioinformatics.

[10] Nguyen L.T., Schmidt H.A., Von Haeseler A., Minh B.Q. (2015). IQ-TREE: A fast and effective stochastic algorithm for estimating maximum-likelihood phylogenies. Mol. Biol. Evol..

[11] Tavaré S., Miura R. (1986). Some Probabilistic and Statistical Problems in the Analysis of DNA Sequences.

[12] Gu X., Fu Y.X., Li W.H. (1995). Maximum likelihood estimation of the heterogeneity of substitution rate among nucleotide sites. Mol. Biol. Evol..

[13] Han A., Parker E., Maurer-Stroh S., Russell C.A. (2019). Inferring putative transmission clusters with Phydelity. Virus Evol..

[14] Li W., Godzik A. (2006). Cd-hit: A fast program for clustering and comparing large sets of protein or nucleotide sequences. Bioinformatics.

[15] Piñeiro C., Abuín J.M., Pichel J.C. (2020). Very Fast Tree: Speeding up the estimation of phylogenies for large alignments through parallelization and vectorization strategies. Bioinformatics.

[16] Lemoine F., Gascuel O. (2021). Gotree/Goalign: Toolkit and Go API to facilitate the development of phylogenetic workflows. NAR Genom. Bioinform..

[17] Foley G., Mora A., Ross C.M., Bottoms S., Sützl L., Lamprecht M.L., Zaugg J., Essebier A., Balderson B., Newell R. (2020). Identifying and engineering ancient variants of enzymes using Graphical Representation of Ancestral Sequence Predictions (GRASP). bioRxiv.

[18] Yu G., Smith D.K., Zhu H., Guan Y., Lam T.T.Y. (2017). GGTREE: An r package for visualization and annotation of phylogenetic trees with their covariates and other associated data. Methods Ecol. Evol..

[19] Hölzer M., Marz M. (2021). PoSeiDon: A Nextflow pipeline for the detection of evolutionary recombination events and positive selection. Bioinformatics.

[20] Kosakovsky Pond S.L., Posada D., Gravenor M.B., Woelk C.H., Frost S.D.W. (2006). GARD: A genetic algorithm for recombination detection. Bioinformatics.

[21] Feng Q., Tiedje K., Ruybal-Pesántez S., Tonkin-Hill G., Duffy M., Day K., Shim H., Chan Y. (2022). A scalable method for identifying recombinants from unaligned sequences. Bioinformatics.

[22] Lam H.M., Ratmann O., Boni M.F. (2018). Improved Algorithmic Complexity for the 3SEQ Recombination Detection Algorithm. Mol. Biol. Evol..

[23] Cerutti G., Guo Y., Zhou T., Gorman J., Lee M., Rapp M., Reddem E.R., Yu J., Bahna F., Bimela J. (2021). Potent SARS-CoV-2 neutralizing antibodies directed against spike N-terminal domain target a single supersite. Cell Host Microbe.

[24] Leman J.K., Weitzner B.D., Lewis S.M., Adolf-Bryfogle J., Alam N., Alford R.F., Aprahamian M., Baker D., Barlow K.A., Barth P. (2020). Macromolecular modeling and design in Rosetta: Recent methods and frameworks. Nat. Methods.

[25] Van Zundert G.C.P., Rodrigues J.P.G.L.M., Trellet M., Schmitz C., Kastritis P.L., Karaca E., Melquiond A.S.J., Van Dijk M., De Vries S.J., Bonvin A.M.J.J. (2016). The HADDOCK2.2 Web Server: User-Friendly Integrative Modeling of Biomolecular Complexes. J. Mol. Biol..

[26] Schymkowitz J., Borg J., Stricher F., Nys R., Rousseau F., Serrano L. (2005). The FoldX web server: An online force field. Nucleic Acids Res..

[27] Vangone A., Bonvin A.M.J.J. (2015). Contacts-based prediction of binding affinity in protein–protein complexes. eLife.

[28] Schrödinger PyMOL. https://pymol.org/.

[29] Planas D., Veyer D., Baidaliuk A., Staropoli I., Guivel-Benhassine F., Rajah M.M., Planchais C., Porrot F., Robillard N., Puech J. (2021). Reduced sensitivity of SARS-CoV-2 variant Delta to antibody neutralization. Nature.

[30] Collier D.A., Marco A.D., Ferreira I.A.T.M., Meng B., Datir R., Walls A.C., Kemp S S.A., Bassi J., Pinto D., Fregni C.S. (2021). SARS-CoV-2 B.1.1.7 escape from mRNA vaccine-elicited neutralizing antibodies. Nature.

[31] Greaney A.J., Loes A.N., Crawford K.H.D., Starr T.N., Malone K.D., Chu H.Y., Bloom J.D. (2021). Comprehensive mapping of mutations in the SARS-CoV-2 receptor-binding domain that affect recognition by polyclonal human plasma antibodies. Cell Host Microbe.

[32] Jungreis I., Sealfon R., Kellis M. (2021). SARS-CoV-2 gene content and COVID-19 mutation impact by comparing 44 Sarbecovirus genomes. Nat. Commun..

[33] Shneider A.D.B., Su M., Hinrichs A., Wang J., Amin H., Bell J., Wadford D.A., O’toole A., Scher E., Perry M.D. SARS-CoV-2 Lineage Assignment Is More Stable with UShER. https://virological.org/t/sars-cov-2-lineage-assignment-is-more-stable-with-usher/781.

[34] Chen Z., Azman A.S., Chen X., Zou J., Tian Y., Sun R., Xu X., Wu Y., Lu W., Ge S. (2022). Global landscape of SARS-CoV-2 genomic surveillance and data sharing. Nat. Genet..

[35] Spiliotopoulos D., Kastritis P.L., Melquiond A.S.J., Bonvin A.M.J.J., Musco G., Rocchia W., Spitaleri A. (2016). dMM-PBSA: A new HADDOCK scoring function for protein-peptide docking. Front. Mol. Biosci..

[36] Gorman J., Rapp M., Kwong P.D., Shapiro L. Cryo-EM Structure of NTD-Directed Neutralizing Antibody 4–8 Fab in Complex with SARS-CoV-2 S2P Spike (RCSB PDB-7LQV). https://www.rcsb.org/structure/7LQV.

[37] Koenig P.A., Das H., Liu H., Kümmerer B.M., Gohr F.N., Jenster L.M., Schiffelers L.D.J., Tesfamariam Y.M., Uchima M., Wuerth J.D. (2021). Structure-guided multivalent nanobodies block SARS-CoV-2 infection and suppress mutational escape. Science.

[38] Huo J., Le Bas A., Ruza R.R., Duyvesteyn H.M.E., Mikolajek H., Malinauskas T., Tan T.K., Rijal P., Dumoux M., Ward P.N. (2020). Neutralizing nanobodies bind SARS-CoV-2 spike RBD and block interaction with ACE2. Nat. Struct. Mol. Biol..

[39] Ahmad J., Jiang J., Boyd L.F., Zeher A., Huang R., Xia D., Natarajan K., Margulies D.H. (2021). Structures of synthetic nanobody–SARS-CoV-2 receptor-binding domain complexes reveal distinct sites of interaction. J. Biol. Chem..

[40] Ge J., Wang R., Ju B., Zhang Q., Sun J., Chen P., Zhang S., Tian Y., Shan S., Cheng L. (2021). Antibody neutralization of SARS-CoV-2 through ACE2 receptor mimicry. Nat. Commun..

[41] Tortorici M.A., Beltramello M., Lempp F.A., Pinto D., Dang H.V., Rosen L.E., McCallum M., Bowen J., Minola A., Jaconi S. (2020). Ultrapotent human antibodies protect against SARS-CoV-2 challenge via multiple mechanisms. Science.

[42] Latif A.A., Mullen J.L., Alkuzweny M., Tsueng G., Cano M., Haag E., Zhou J., Zeller M., Hufbauer E., Matteson N. B.1.1.523 Lineage Report. https://outbreak.info/situation-reports?pango=B.1.1.523.

[43] McCallum M., Walls A.C., Sprouse K.R., Bowen J.E., Rosen L., Dang H.V., deMarco A., Franko N., Tilles S.W., Logue J. (2021). Molecular basis of immune evasion by the delta and kappa SARS-CoV-2 variants. Science..

[44] Wise J. (2021). COVID-19: The E484K mutation and the risks it poses. BMJ.

[45] Liu Z.V., VanBlargan L.A., Bloyet L.M., Rothlauf P.W., Chen R.E., Stumpf S., Zhao H., Errico J.M., Theel E.S., Liebeskind M.J. (2021). Identification of SARS-CoV-2 spike mutations that attenuate monoclonal and serum antibody neutralization. Cell Host Microbe.

[46] Lassaunière R., Polacek C., Fonager J., Bennedbæk M., Boding L., Rasmussen M., Fomsgaard A. (2021). Neutralisation of the SARS-CoV-2 Delta variant sub-lineages AY.4.2 and B.1.617.2 with the mutation E484K by Comirnaty (BNT162b2 mRNA) vaccine-elicited sera, Denmark, 1 to 26 November 2021. Eurosurveillance.

[47] Li T., Cui Z., Jia Y., Liang Z., Nie J., Zhang L., Wang M., Li Q., Wu J., Xu N. (2022). Aggregation of high-frequency RBD mutations of SARS-CoV-2 with three VOCs did not cause significant antigenic drift. J. Med. Virol..

[48] Baj A., Novazzi F., Pasciuta R., Genoni A., Ferrante F.D., Valli M., Partenope M., Tripiciano R., Ciserchia A., Catanoso G. (2021). Breakthrough Infections of E484K-Harboring SARS-CoV-2 Delta Variant, Lombardy, Italy. Emerg. Infect. Dis..

[49] Hu J., Peng P., Cao X., Wu K., Chen J., Wang K., Tang N., Huang A. (2022). Long increased immune escape of the new SARS-CoV-2 variant of concern Omicron. Cell. Mol. Immunol..

[50] Kisa S., Kisa A. (2020). Under-reporting of COVID-19 cases in Turkey. Int. J. Health Plan. Manag..

[51] Gowthaman R., Guest J.D., Yin R., Adolf-Bryfogle J., Schief W.R., Pierce B.G. (2021). CoV3D: A database of high resolution coronavirus protein structures. Nucleic Acids Res..

