# Supplementary material for: Determining the International Spread of B.1.1.523 SARS-CoV-2 Lineage with a Set of Mutations Highly Associated with Reduced Immune Neutralization
Source: Microorganisms. 2022 Jul 5;10(7):1356. doi: 10.3390/microorganisms10071356 (PMC9320768; doi:10.3390/microorganisms10071356)
Supplement: Supplementary file 1 [file Figure S1.pdf › Figure S1.pdf]

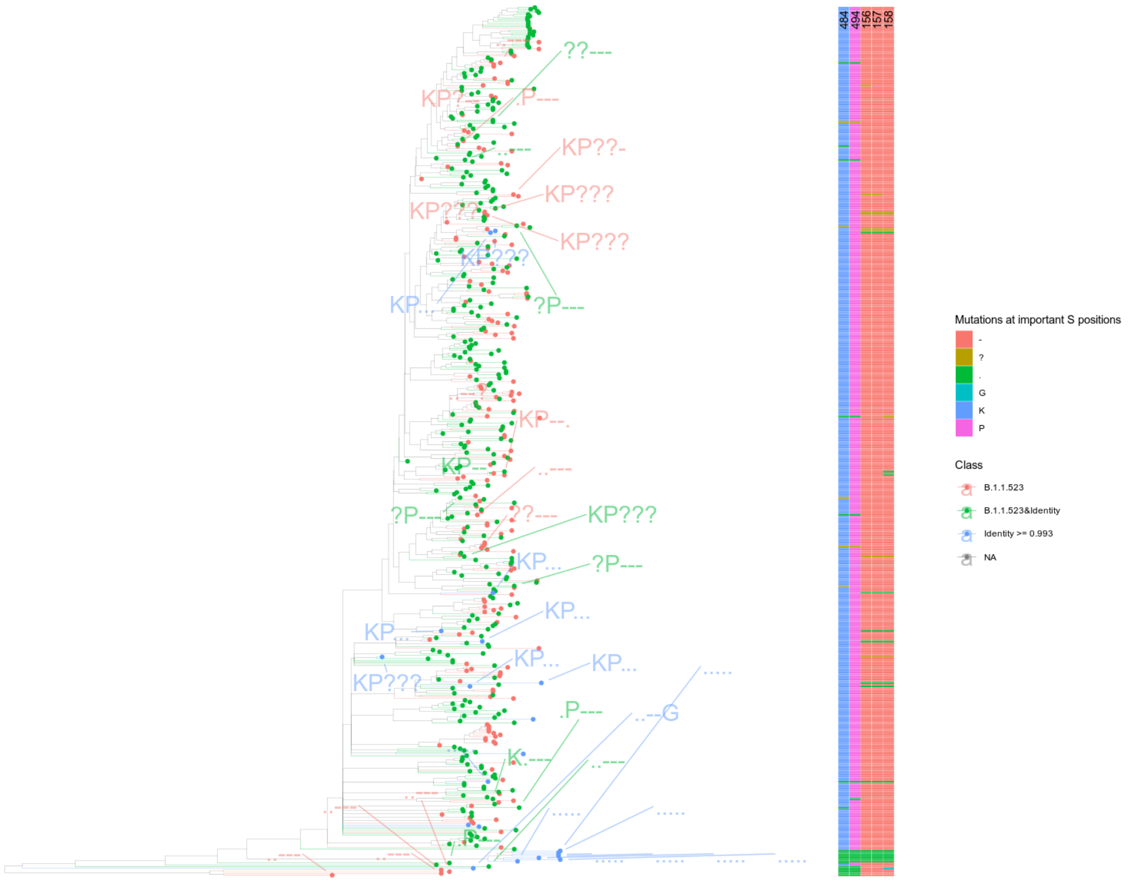

Figure S1. Maximum likelihood tree based on the genomic sequences that are classifiable as B.1.1.523 lineage together with the closest sequences based on identity search. The heatmap indicates residues at the 484,494 and 156-158 positions. The five letter length labels indicate cases and corresponding haplotypes where the residues do not match the expected ones for the B.1.1.523 lineage.
